# Continuous Flow Esterification of a *H*-Phosphinic Acid, and Transesterification of *H*-Phosphinates and *H*-Phosphonates under Microwave Conditions

**DOI:** 10.3390/molecules25030719

**Published:** 2020-02-07

**Authors:** Nóra Zsuzsa Kiss, Réka Henyecz, György Keglevich

**Affiliations:** Department of Organic Chemistry and Technology, Budapest University of Technology and Economics, 1111 Budapest, Hungary; zsnkiss@mail.bme.hu (N.Z.K.); henyecz.reka@mail.bme.hu (R.H.)

**Keywords:** *H*-phosphinic acid, esterification, *H*-phosphinates, *H*-phosphonates, transesterification, microwave flow reactor

## Abstract

The microwave (MW)-assisted direct esterification of phenyl-*H*-phosphinic acid, transesterification of the alkyl phenyl-*H*-phosphinates so obtained, and the similar reaction of dibenzyl phosphite (DBP) were investigated in detail, and the batch accomplishments were translated into a continuous flow operation that, after optimization of the parameters, such as temperature and flow rate, proved to be more productive. Alcoholysis of DBP is a two-step process involving an intermediate phosphite with two different alkoxy groups. The latter species are of synthetic interest, as precursors for optically active reagents.

## 1. Introduction

It has been a great challenge in the pharmaceutical industry to transform batch realizations of organic chemical reactions into continuous flow methods [1,2,3]. Kappe is one of the most prominent chemists who have elaborated flow chemical accomplishments that are welcome by the pharmaceutical industry in order to introduce up to date techniques, in the first approach, in the R&D segment [1]. However, the “sine qua non” of the realization of the flow techniques is that the mixtures should be homogenous and non-viscous that represents a limitation. Due to the dynamical development in the field, up-to-date models of MW reactors have appeared on the market. The application of the MW technique embraces above all organic chemical syntheses, the preparation of nanomaterials, and broadly understood material processing [4,5,6]. The most suitable reactions for MW assistance include multicomponent reactions, condensations, eliminations, and substitutions as exemplified by esterifications, C–C cross couplings, dehydrations and the Mannich condensation [7]. The combination of the flow technique with MW irradiation represents a big step further, as it broadens the sphere of reactions that can be performed [8]. We have had interests in converting batch MW-promoted reactions involving organophosphorus transformations into flow operation [9,10,11]. Ionic liquids (ILs) are regarded as green solvents [12]. However, there is a vision that ILs might cause a real breakthrough as additives or catalysts [13,14].

P-esters, like phosphinates and phosphonates may be important building blocks in synthetic organic chemistry [15,16]. *H*-Phosphinates and *H*-phosphonates are typical starting materials for the Hirao P–C coupling reactions and the Kabachnik–Fields condensations resulting in the formation of aryl-phosphinates/phosphonates and α-aminophosphonates, respectively [17,18,19]. α-Amino-phosphonates are important due to their potential biological activity connected to their enzyme inhibitory effect. A novel preparation of phosphinates involves the microwave (MW)-assisted direct esterification of phosphinic acids with alcohols [20,21,22]. The similar esterification of phosphonic acids was a more difficult task [23]. We found that a suitable IL additive may promote the esterification of phosphinic acids [24,25], and the monoesterification of phosphonic acids [25]. It was found that alkylation was more suitable to convert the second hydroxy group into an alkoxy unit [26]. The senior author of this article together with co-workers developed the MW-assisted transesterification (alcoholysis) of dialkyl phosphites [27,28]. It was possible to conduct the reactions to afford the dialkyl phosphites with two different alkyl groups as the predominating products.

In this article we summarize our experience acquired during the translation of batch MW preparations of *H*-phosphinates and *H*-phosphonates into flow processes. Esterifications and transesterifications were chosen as suitable model reactions, as, in these cases, MW irradiation and IL additives proved to be useful in our earlier studies.

## 2. Results and Discussion

### 2.1. MW-Assisted Direct Esterification of Phenyl-H-Phosphinic Acid *(**1**)*

Before the flow chemical attempts, let us survey the precedents on the batch MW synthesis of alkyl phenyl-*H*-phosphinates (**2**). In the first round, phosphinic acid **1** was reacted with ethyl and other linear or branched C_3_–C_5_ and C_8_ alcohols applied in a 15-fold quantity at 160–200 °C to afford the esters **2a**, **2b**, **2d**–**i** in yields of 73–90% (Table 1/Entries 1, 3, 7–9, 11, 13, 15, 17 and 19). More developed syntheses were performed in the presence of 10% of [bmim][PF_6_] at a lower temperature of 140–160 °C providing the products **2a**, **2b**, **2d**–**I** after a short reaction time of 30 min in somewhat higher yields of 82–94% (Table 1/Entries 2, 4, 10, 12, 14, 16, 18 and 20). It was found earlier that a catalytic amount (5–10%) of the IL is beneficial in the direct esterifications. A few ILs were tested as additives. Although all tested ILs enhanced the esterifications, [bmim][PF_6_] was the best one [24]. In the small-scale reactions it was appropriate to apply 10% of the IL. The basic role of the IL additive may be to enhance the absorption of MWs due to its polar nature. The results with *i*-propanol referred to steric hindrance, as an almost complete conversion could only be attained at 180 °C in the presence of the IL (Table 1/Entries 5 and 6). Most of the results were reported earlier [20,26] that were completed by a few new data (Table 1/Entries 4, 6, 8, 9, 15, 16, 19 and 20).

**Figure 1:**
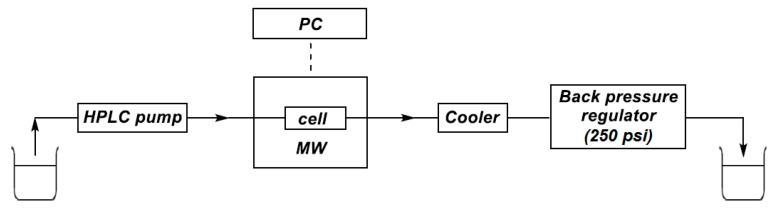
Sketch of the continuous flow system used.

**Figure 2:**
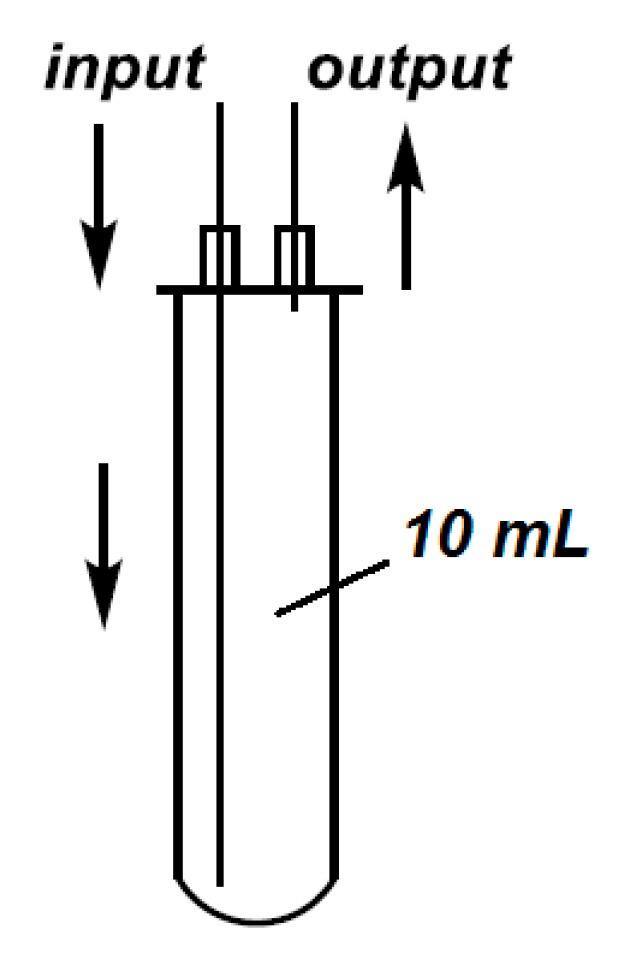
The commercial continuous flow cell.

Next, we tried to convert the esterification into a flow method. The sketch of the continuous flow system used in our experiments is shown in Figure 1. A commercially available flow cell (Figure 2) was inserted into the CEM reactor, and the transport of the PhP(O)H(OH)/ROH mixture was ensured by a HPLC pump. The pressure was maintained by a back pressure regulator.

During the continuous flow esterification of phenyl-*H*-phosphinic acid (**1**), 0.10 g **1**/mL alcohol solutions were prepared, and fed in the reactor at different temperatures (160–200 °C) and flow rates (Table 2). The unstationary phase that was comparable with the residence time (at V = 0.15 mL/min and V =0.25 mL/min t = 67 min and t = 40 min, respectively) was followed by the steady state operation. The esterifications were monitored by ^31^P-NMR measurements. The reaction of phosphinic acid **1** with ^n^BuOH was investigated in detail. In this particular case, the 0.1 g/mL concentration means 15-fold quantity of the alcohol. Increasing the temperature from 160 °C to 180 °C, and then to 200 °C, at a flow rate of 0.25 mL/min, the conversions were 50%, 53% and 63%, respectively (Table 2/Entries 1, 3 and 5). At the same temperatures, but setting a lower flow rate of 0.15 mL/min that allows a longer residence time in the reactor, somewhat higher conversions of 54%, 64% and 72%, respectively, were detected (Table 2/Entries 2, 4 and 6). The addition of 5% of [bmim][PF_6_] to the mixture of the reagents prior to irradiation was helpful to attain higher conversions. It has to be mentioned that 5% of the IL was sufficient. Applying a flow rate of 0.25 mL/min at 160 °C, 180 °C and 200 °C, the conversions were 66%, 83% and 100%, respectively (Table 2/Entries 7, 9 and 11). At a lower rate of 0.15 mL/min, the conversions were somewhat higher 72% (160 °C) and 95% (180 °C) (Table 2/Entries 8 and 10) than setting 0.25 mL/min. In the next step, the volatile alcohols EtOH, ^n^PrOH and ^i^PrOH were reacted at the possible maximum temperatures of 160–180 °C applying the lower flow rate of 0.15 mL/min. In these cases, the conversions were 65%, 71% and 68%, respectively (Table 2/Entries 12–14). Recycling the mixture from the esterification with EtOH, and re-reacting it under the same conditions (160 °C/0.15 mL/min), the conversion became quantitative (see footnote “e” of Table 2). The comparative thermal esterification of phosphinic acid **1** with EtOH at 160 °C applying a flow rate of 0.15 mL/min proceeded until a conversion of 35% (see footnote “d” of Table 2). Using ^i^BuOH (160 °C, 0.15 mL/min), the conversion was quantitative (Table 2/Entry 15). ^n^PentOH, ^i^PentOH, ^n^OctOH and ^i^OctOH allowed the application of a somewhat higher temperature of 180–200 °C. In these cases, the higher rate of 0.25 mL/min was efficient at 190 °C (and in one case at 200 °C) as the conversions were quantitative (Table 2/Entries 17, 19, 21 and 23). Applying a lower flow rate of 0.15 mL/min at somewhat lower temperature of 180 °C, the conversion was 100%, or almost quantitative (Table 2/Entries 16, 18, 20 and 22). The yields of the phosphinates **2a**–**i** prepared from the best experiments fell in the range of 63–91%. If there is a time frame for the preparation of the esters (**2**), it is worth choosing the parameter set of 190 °C/0.25 mL/min against 180 °C/0.15 mL/min.

Comparing the batch and continuous flow preparation of the butyl-(**2d**) or pentyl phosphinate (**2f**) (Table 1/Entries 10 and 14 vs. Table 2/Entries 11 and 17), one can conclude that the flow operation afforded products **2d** and **2f** in a 4.5-fold and 6.9-fold higher quantity, respectively, as compared to the corresponding batch method. Of course, during the comparison, the operation time of the flow reactor should be equal to the reaction time applied in the batch reactor. It can be said that the batch method provides ca. 0.10 g ester/30 min, while the flow preparation may give ca. 0.75 g product after the same time. It can be concluded that the batch approach is more limited in respect of scale. If more alkyl phenyl-*H*-phosphinate is needed, it is worth choosing the flow operation. It is noteworthy that the quantity of the IL (that is the most expensive component) could be halved, as 5% was enough.

### 2.2. MW-Assisted Transesterification of Ethyl-Phenyl-H-Phosphinate *(**2a**)*

As an alternative method to direct esterification, transesterification (alcoholysis) is another option for the preparation of esters, and seemed to be a suitable model for MW application. For this, we wished to investigate the reaction of ethyl phenyl-*H*-phosphinate (**2a**) (a commercially available P-ester) with simple alcohols under MWs to prepare other representatives of this family of compound. The C_1_, C_3_–C_5_ alcohols, along with BnOH were applied in a 15-fold quantity, and with the exception of the volatile MeOH, they were used at 160–190 °C. The experimental data are listed in Table 3. One can see that in reaction with MeOH at 120 °C for 3 h and at 140 °C for 2 h, a conversion average of 91% was attained (Table 3/Entries 1 and 2). Alcoholysis with ^n^PrOH and ^i^PrOH at 180 °C took place in conversions of 97% and 89%, after reaction times of 1 h and 2 h, respectively (Table 3/Entries 3 and 4). Regarding ^n^BuOH, quantitative conversions could be observed at parameter sets of 160 °C/2.25 h and 180 °C/40 min (Table 3/Entries 5 and 6). The transesterifications of ethyl phosphinate **2a** with ^i^BuOH, ^n^PentOH, ^i^PentOH, 3-PentOH and BnOH were complete at 160 °C/2.25 h, 180 °C/40 min, 190 °C/40 min, 190 °C/45 min, and 180 °C/1 h, respectively (Table 3/Entries 7–11). Phosphinates **2b**–**g, 2j**–**l** were obtained in yields of 74–91% after flash column chromatography. One may conclude that the uncatalyzed transesterifications of *H*-phosphinate **2a** requires harsh conditions, but can be performed efficiently under MW irradiation.

In the next phase, we tried to elaborate the continuous flow transesterification of ethyl phosphinate **2a** with ^n^BuOH applied in a 15-fold excess quantity. As can be seen from Table 4, at 180 or 200 °C, the alcoholysis remained incomplete (as characterized by conversions of 53–84%) no matter if the flow rate was 0.25 or 0.15 mL/min (Table 4/Entries 1–4). At 220 °C, the conversions were 81% (0.25 mL/min) and 94% (0.15 mL/min) (Table 4/Entries 5 and 6). The optimum parameter set for a quantitative reaction involved a temperature of 225 °C and a flow rate of 0.15 mL/min (Table 4/Entry 7). In this case, the yield of butyl phosphinate **2d** was 85%. Adopting these parameters to the transesterification of phosphinate **2a** with ^i^BuOH, ^n^PentOH and ^i^PentOH, the corresponding esters **2e**–**g** were obtained in complete conversions, and in high yields of 82–89%.

### 2.3. MW-Assisted Transesterification of Dibenzyl Phosphite *(**3**)*

Keglevich and co-workers have investigated the MW-assisted transesterifications (alcoholyses) of dialkyl phosphites [27,28]. These kinds of reactions take place in two steps resulting first in a phosphite with two different alkyl groups, and in the second step the fully transesterified dialkyl phosphite. The outcome of the reaction depended on the temperature, and on the molar ratio of the reactants. It was not easy to achieve selectivity. At the same time, it is known that the benzyl phosphonates may undergo easy substitution of the BnO group [29]. For this, alcoholysis of dibenzyl phosphite (**3**) seemed to be an appropriate model. Simple C_1_–C_4_ alkyl alcohols were used as reactants in 25 equivalent quantities in the temperature range of 80–130 °C under MW irradiation. Experimental data can be found in Table 5. In reaction with MeOH, irradiation at 80 °C for 3 h or at 120 °C for 0.5 h led to similar results, to a mixture containing 26/26% starting phosphite **3**, 57/54% of the intermediate **4a**, and 17/20% of the fully transesterified phosphite **5a** (Table 5/Entries 1 and 3). Running the alcoholysis at 100 °C for 2 h or at 120 °C for 1.5 h, dimethyl ester **5a** predominated in 56/70% (Table 5/Entries 2 and 4). After a 2.5 h heating the diester (**5a**) was present in a maximum quantity of 87% (Table 5/Entry 5). Using EtOH, the course of alcoholysis towards diethyl phosphite was somewhat slower than that with MeOH (Table 5/Entries 6, 9 and 10 vs. 1, 2 and 3, respectively). After an irradiation at 120 °C for 1 h, the ratio of products **3b**, **4b** and **5b** was 9:51:40, that after 4 h was shifted to 0:11:89 (Table 5/Entries 11 and 12). A comparison was made at 100 °C/0.5 h to see the effect of 20% of [bmim][PF_6_] as an additive. In the absence of the IL, the starting dibenzyl phosphite (**3**) was the main component (65%), while performing the alcoholysis in the presence of the additive, the diethyl ester (**5**) predominated (58%) (Table 5/Entries 7 and 8). In the presence of ^i^PrOH as the agent, the consecutive transformation was slower at 100 and 120 °C (Table 5/Entries 13 and 14). There was need for a 5 h irradiation at 130 °C to compensate the effect of steric hindrance (Table 5/Entries 15 and 16). In reaction with ^n^BuOH, almost similar results were obtained as with EtOH (Table 5/Entries 17, 18 and 20 vs. 9, 10 and 12). A comparative thermal experiment at 100 °C for 2 h took place in a lower conversion of 73% (Table 5/Entry 17/ footnote “d”). It is recalled that the conversion of the MW variation was 92% (Table 5/Entry 17). While the relative quantity of the intermediate (**4d**) was almost the same (59/61%), that of dibutyl phosphite (**5d**) was 14% (Δ) and 31% (MW).

It is noteworthy that the valuable *H*-phosphonates with different alkyl groups could be obtained in a maximum proportion of 57% (**4a**), 68% (**4b**), 60% (**4c**) and 61% (**4d**) covered by entries 1, 10, 13 and 17, respectively (Table 5). Isolated yields of the BnO–RO phosphonates **4a**–**d** fell in the range of 47–59%.

Regarding the conditions (T and t) needed to reach complete conversions (disappearance of the starting material (**3**) from the mixture, and predominant appearance of the fully transesterified product (**5**)) (see entries 5, 12, 16 and 20 of Table 5), the order of reactivity of the alcohols was the following: MeOH > BuOH ~ EtOH > ^i^PrOH.

**Figure 3:**
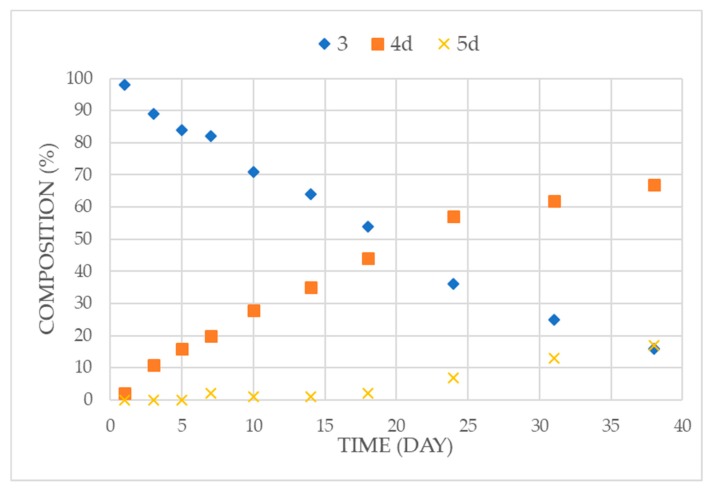
Alcoholysis of dibenzyl phosphite (**3**) with butanol at room temperature.

It is worth noting that dibenzyl phosphite (**3**) is significantly more reactive in transeserifications than ethyl phenyl-*H*-phosphinate **2a**. The enhanced reactivity of dibenzyl phosphite (**3**) in transesterification prompted us to try the reaction with ^n^BuOH at room temperature. The data summarized in Table 6 and Figure 3. showed that the consecutive transesterification took place slowly: after 18 days, there was 54% of the starting phosphite (**3**) together with 44% of the “mixed” ester **4d**, and 2% of the dibutyl derivative **5d** (Table 6/Entry 7). The final “equilibrium” concentration was attained after 38 days, when the mixture comprised 16% of the starting material (**3**), 67% of the Bu-Bn ester (**4d**) and only 17% of the dibutyl ester (**5d**) (Table 6/Entry 10). This experiment was found reproducible. It is assumed that the application of a larger excess of BuOH would result in the shift of the equilibrium toward esters **4d** and **5d**. However, it is worth noting that the composition of the above “equilibrium” mixture with 67% of the benzyl-butyl ester (**4d**) is favorable, as it is a valuable intermediate.

The next step was to try the continuous flow method. The transesterification of dibenzyl phosphite (**3**) with MeOH at 110 °C applying a flow rate of 0.25 mL/min led to a mixture containing 24% of the starting material (**3**), 52% of the “mixed” ester (**4a**) and 24% of dimethyl phosphite (**5a**) (Table 7/Entry 1). At 120 °C, the composition was 17% (**3**), 44% (**4a**) and 39% (**5a**) (Table 7/Entry 2). Operation at a lower rate of 0.15 mL/min and at 135 °C provided the three components (**3a**, **4a** and **5a**) in relative quantities of 5%, 23% and 72%, respectively (Table 7/Entry 3). EtOH displayed somewhat lower reactivity, and under the previous two sets of parameters, mixtures containing 28% of **3b**, 48% of **4b**, 24% of **5b**, and 7% of **3b**, 27% of **4b** and 66% of **5b**, respectively, were obtained (Table 7/Entries 4 and 7). The use of parameter sets of 0.15 mL/min at 120 °C and 0.25 mL/min at 135 °C resulted in a comparative outcomes of 20/17% of **3b**, 40/36% of **4b** and 40/47% of **5b** (Table 7/Entries 5 and 6). In agreement with expectation, ^i^PrOH was found to be the less reactive alcohol. Setting a flow rate of 0.25 mL/min at 120 °C, the composition of the reaction mixture was 49% of **3c**, 48% of **4c** and 3% of **5c** (Table 7/Entry 8). In order to achieve a more complete conversion, a temperature of 145 °C and a rate of 0.15 mL/min were applied (Table 7/Entry 9). The results with ^n^BuOH were again rather similar to those obtained with EtOH (Table 7/Entries 10 and 11 vs. entries 4 and 7). The experiments providing the phosphites with different alkoxy groups **4a**–**d** are of importance, as the “mixed” phosphites may be used as valuable intermediates in the reactions outlined in the Introduction. Optical resolution may lead to enantiomer-enriched forms of the >P(O)H species. The best runs are marked by entries 1, 4, 8 and 10 of Table 7. The proportions of 47–52% allowed isolated yields of 39–44% for the “mixed” esters **4a**–**d**. A comparative thermal transesterification of dibenzyl ester **3** with butanol at 120 °C and at a flow rate of 0.25 mL/min led to a composition of 49% of starting material **3**, 47% of benzyl-butyl ester **4d**, and 4% of dibutyl ester **5d**, suggesting that on conventional heating, the efficiency is lower (compare footnote “d” of Table 7 with Entry 10).

As a novel trial, the pre-reacted mixture of dibenzyl phosphite (**3**) and BuOH (26 °C, 18 days) comprising 55% of dibenzyl phosphite, 41% of the “mixed” ester (**4d**) and 4% of dibutyl phosphite (**5d**) was re-fed into the flow reactor at 120 °C applying 0.25 mL/min. The final mixture contained 8% of the starting material (**3**), as well as 23% and 69% of esters **4d** and **5d**, respectively. Hence, the product ratio could be shifted towards the fully transesterified product **5d**.

## 3. Materials and Methods

### 3.1. General Information

The MW-assisted reactions were carried out in a Discover (300 W) focused MW reactor (CEM Microwave Ltd. Buckingham, UK) equipped with a stirrer and a pressure controller applying irradiation. The reaction temperature was monitored by an external IR sensor located at the bottom of the cavity.

The continuous flow reactions were performed in a system using a CEM^®^ Discover (300 W) focused MW reactor equipped with a CEM^®^ 10-mL Flow Cell Accessory continuous flow unit (irradiated volume 7 mL). The material flow was ensured by a 305 HPLC pump (Gilson Inc., Middleton, WI, USA) while the pressure of 250 psi (17.2 bar) was maintained by an HPLC backpressure regulator. Teflon^®^ tubes with an outside diameter of 3.175 mm and an inside diameter of 1.575 mm were used. All of the accessories applied were compatible with a regular HPLC system. The reaction temperature was monitored by an external IR sensor.

The ^31^P-, ^13^C- and ^1^H-NMR spectra were obtained in CDCl_3_ or DMSO-*d*_6_ solution on an AV-300 or DRX-500 spectrometer (Bruker AXS GmBH, Karlsruhe, Germany) operating at 121.5, 75.5 and 300 or 202, 126 and 500 MHz, respectively. The ^31^P-NMR chemical shifts are referred to H_3_PO_4_, while the ^13^C and ^1^H chemical shifts to TMS. The couplings are given in Hz. The exact mass measurements were performed using an 6545 QTOF mass spectrometer (Agilent Technologies, Santa Clara, CA, USA) in high resolution, positive electrospray mode.The reagents and solvents were purchased from Sigma-Aldrich Ltd. (St. Louis, MO, USA), and were used as received without further purification.

The product ratios were determined on the basis of relative ^31^P-NMR intensities. Representative examples are shown in the Supplementary Materials for the cases marked by Table 2/Entries 5 and 11, Table 3/Entry 7 and Table 5/Entry 13.

### 3.2. General Procedure for the Batch Esterificaton of Phenyl-H-Phosphinic Acid *(**1**)*

A mixture of phosphinic acid **1** (0.10 g, 0.70 mmol) and 15 equivalents of the alcohol (0.60 mL of ethanol, 0.79 mL of *n*-propanol, 0.80 mL of isopropanol, 0.96 mL of *n*-butanol, 1.0 mL of isobutanol, 1.14 mL of *n*-pentanol, 0.14 mL of *i*-pentanol, 1.65 mL of *n*-octanol, 1.64 mL of 2-ethylhexanol) was measured into a sealed tube in the presence or absence of 13.6 μL (0.07 mmol) of [bmim][PF_6_], and irradiated in a CEM MW reactor at first with a power of 200–300 W, and after the set temperature was attained, it was maintained by an automatic regulation using 80–150 W. The values for the temperature and pressure together with the times are shown in Table 1. Then, the alcohol was removed under reduced pressure, and the residue so obtained purified by flash column chromatography using silica gel and ethyl acetate as the eluent to give phosphinates **2** as oils in a purity of ≥98% according to GC. Identification data of the phosphinates **2a**–**i** can be found in Table 8.

### 3.3. General Procedure for the Continuous Flow Direct Esterification of Phenyl-H-Phosphinic Acid *(**1**)*

A mixture of phosphinic acid **1** (10.0 g, 70.4 mmol) and 100 mL of an alcohol (2.8 mol of ethanol, 2.1 mol of n-propanol, 2.1 mol of isopropanol, 1.1 mol of *n*-butanol, 1.1 mol of *i*-butanol, 0.92 mol of *n*-pentanol, 0.92 mol of *i*-pentanol, 0.64 mol of *n*-octanol, 0.64 mol of 2-ethylhexanol) was homogenized by stirring for 5 min at 25 °C in the presence or absence of 3.5 mmol (0.68 mL) of [bmim][PF_6_]. The reactor was flushed with 20 mL of the mixture with a flow rate of 10 mL/min at 25 °C and 17 bar. Then, the flow rate was set to the desired value (see Table 2), and the flow cell was irradiated with a power of 200–300 W for a few minutes, until the desired temperature was reached. After this, the power was controlled automatically (by 80–150 W) to maintain the value set. The operation was regarded steady state after an unstationary phase of 45–70 min as suggested by ^31^P- NMR analysis. After a 45 min or 75 min period of steady state operation belonging to 0.25 mL/min and 0.15 mL/min, respectively, the collected sample was concentrated under reduced pressure, and the residue so obtained purified by flash column chromatography using silica gel and ethyl acetate as the eluent to afford phosphinates **2** as oils in a purity of ≥98% according to GC.

The yields were calculated on the basis of the molar quantity of the ester [m(**2**)/Mw(**2**), where m(**2**): weight of ester **2**, Mw(**2**): molecular weight of ester **2**] obtained after purification, taking into consideration the molar quantity of the phosphinic acid fed in during the given time [V∙t∙c(**1**), where V: the flow rate (0.15 mL/min or 0.25 mL/min), t: time of stationary operation (45 min or 75 min) and c(**1**): molar concentration of acid **1** (0.65 mmol/mL)]

Realization of the recirculation: the reaction mixture obtained from 10.0 g (70.4 mmol) of phosphinic acid **1** and 100 mL (2.8 mol) of ethanol in the presence of 0.68 mL (3.5 mmol) of [bmim][PF_6_] as shown above in the general procedure (and marked by Table 2/Entry 12) was placed in the container of the starting mixture, and after a flush with 20 mL of the mixture with a flow rate of 10 mL/min at 25 °C and 17 bar, and after setting a flow rate of 0.15 mL/min, the temperature was adjusted to 160 °C exactly as described above for the first run. The product was collected after getting in the stationary operation phase (Table 2/footnote “e”).

### 3.4. General Procedure for the Batch Transesterification of Ethyl-H-Phenylphosphinate *(**2a**)*

A mixture of ethyl-*H*-phenylphosphinate (**2a**, 0.10 g, 0.58 mmol) and 15 equivalents of an alcohol (0.36 mL of methanol, 0.66 mL of *n*-propanol, 0.70 mL of isopropanol, 0.81 mL of *n*-butanol, 0.81 mL of *i*-butanol, 0.96 mL of *n*-pentanol, 0.96 mL of *i*-pentanol, 0.95 mL of 3-pentanol, 0.92 mL of benzyl alcohol) was measured in a sealed tube and irradiated in the MW reactor at first with a power of 80–300 W, and after the set temperature was attained, it was maintained by an automatic regulation using 50–150 W. The temperatures and the times are shown in Table 3. Then, the alcohol was removed under reduced pressure, and the residue so obtained purified by flash column chromatography using silica gel and ethyl acetate as the eluent to afford phosphinates **2** as oils in a purity of ~98% according to GC.

### 3.5. General Procedure for the Continuous Flow Transesterification of Ethyl-H-Phenylphosphinate *(**2a**)*

A mixture of ethyl-*H*-phenylphosphinate (**2a**, 10.0 g, 58.8 mmol) and 100 mL of an alcohol (1.1 mol of *n*-butanol, 1.1 mol of *i*-butanol, 0.92 mol of *n*-pentanol and 0.92 mol of *i*-pentanol) was homogenized by stirring for 5 min at 25 °C. The reactor was flushed with 20 mL of the mixture with a flow rate of 10 mL/min at 25 °C and 17 bar. Then, the flow rate was set to the desired value (see Table 4), and the system irradiated as described above under point 3 (300 W/150–200 W). The operation was regarded steady state on the basis of the ^31^P-NMR analyses. In the preparative experiments, the solutions containing esters **2d**–**g** were collected for 1 h. Excess of the alcohol of the collected fraction was removed under reduced pressure, and the residue so obtained purified by flash column chromatography using silica gel and ethyl acetate as the eluant to provide phosphinates **2d**–**g** as oils in a purity of ~98% according to GC. The yield of **2d**–**g** was calculated similarly as shown for that of **2a**–**i** above [(m(**2**)/Mw(**2**)/ V∙t∙c(**2a**), where V: 0.15 mL/min, t: 1 h, c(**2a**): 0.54 mmol/mL.

### 3.6. General Procedure for the Batch Transesterification of Dibenzyl Phosphite

A mixture of dibenzyl phosphite (0.11 mL, 0.50 mmol) and 12.5 mmol of an alcohol (0.51 mL of methanol or 0.73 mL of ethanol or 0.96 mL of isopropanol or 1.1 mL of butanol) was heated in a sealed tube in the MW reactor (at first with a power of 60–80 W, then, in the maintainance phase by 30–50 W) at the temperatures and for the times shown in Table 5. The volatile components were removed under vacuum, and the residual oil was analyzed by ^31^P-NMR spectroscopy. The crude mixture was purified by column chromatography using hexane–ethyl acetate 6:4 (for **4a**–**c**) or hexane–acetone 8:2 (for **4d**) as the eluent. The phosphites **4a**–**d** with different alkoxy groups were obtained as colorless oils in >99% purities.

### 3.7. General Procedure for the Continuous Flow Transesterification of Dibenzyl Phosphite

A mixture of dibenzyl phosphite (7.7 mL, 35 mmol) and 0.88 mol of an alcohol (35.6 mL of methanol or 51.4 mL of ethanol or 67.8 mL of isopropanol or 80.5 mL of butanol) was homogenized by stirring for 5 min at 25 °C. The reactor was flushed with 20 mL of the mixture with a flow rate of 10 mL/min at 25 °C and 17 bar. Then, the flow rate was set to the desired value (see Table 7), and the flow cell was irradiated with a power of 60-100 W for a few minutes, until the desired temperature was reached. After this, the power was controlled automatically (by 30–70 W) to maintain the value set. The operation was regarded steady state on the basis of the ^31^P-NMR results. Development of the steady state condition required ca. 45–80 min. The details can be seen in Table 7. The solutions containing esters **4a**–**d** were collected until 30 min (0.25 mL/min) or 45 min (0.15 mL/min). The volatile components of the collected fractions were removed in vacuum, and the crude mixture was purified by column chromatography as above (see Section 3.6). Products **4a**–**d** were obtained as colorless oils in >99% purities. Yields were calculated on the basis of the isolated molar quantity of the product **4a**–**d** as compared to the molar quantity of dibenzyl phosphite (**3**) fed in during the stationary operation. 

Realization of the experiment starting from a pre-reacted mixture: the pre-reacted mixture of dibenzyl phosphite (3, 7.1 mL, 32.0 mmol) and BuOH (73.2 mL, 0.80 mol) obtained at 26 °C after a 18 days stirring was fed in the MW reactor at 120 °C at a flow rate of 0.25 mL/min as the “fresh” mixtures exactly as shown above to result in a mixture consisting 8% of **3**, 23% of the ”mixed” ester **4d**, and 69% of the fully transesterified product **5d** in the stationary phase.

### 3.8. Product Characterization Data

*Benzyl methyl-H-phosphonate* (**4a**). Yield: 0.50 g (44%); ^31^P-NMR (CDCl_3_) δ 9.2; ^13^C-NMR (CDCl_3_) δ 52.1 (d, *J* = 5.9, OCH_2_^a^), 67.5 (d, *J* = 5.6, OCH_3_^a^), 128.1 (C_2_^b^), 128.8 (C_3_^b^ and C_4_), 135.7 (d, *J* = 5.9, C_1_); ^1^H-NMR (CDCl_3_) δ 3.71 (d, *J* = 12.0, 3H, OCH_3_), 5.11 (d, *J* = 9.7, 2H, OCH_2_), 6.84 (d, *J* = 703.0, 1H, PH), 7.32–7.42 (m, 5H, ArH), ^a,b^may be reversed; [M + H]^+^ = 187.0528, C_8_H_12_O_3_P requires 187.0524.

*Benzyl ethyl-H-phosphonate* (**4b**). Yield: 0.36 g (41%); ^31^P-NMR (CDCl_3_) δ 8.6; δ_P_ [31] (CDCl_3_) 5.2; ^13^C-NMR (CDCl_3_) δ 16.4 (d, *J* = 6.3, CH_3_), 62.1 (d, *J* = 5.9, OCH_2_), 67.3 (d, *J* = 5.5, OCH_2_), 128.1 (C_2_*), 128.7 (C_4_), 128.8 (C_3_*) 135.8 (d, *J* = 6.0, C_1_), *may be reversed; ^1^H-NMR (CDCl_3_) δ 1.31 (t, *J* = 7.1, 3H, CH_3_), 4.00–4.19 (m, 2H, OC*H*_2_CH_3_), 5.10 (d, *J* = 9.6, 2H, OC*H*_2_Ph), 6.86 (d, *J* = 700.0, 1H, PH), 7.30–7.42 (m, 5H, ArH); δ_H_ [31] 1.31 (t, 3H, *J* = 7.0), 4.09 (qd, 2H, *J*_1_ = 7.0, *J*_2_ = 9.2), 5.10 (d, 2H, *J* = 9.6), 6.86 (d, 1H, *J* = 698.9), 7.37 (m, 5H); [M + H]^+^ = 201.0685, C_9_H_14_O_3_P requires 201.0681.

*Benzyl isopropyl-H-phosphonate* (**4c**). Yield: 0.29 g (39%); ^31^P-NMR (CDCl_3_) δ 6.2; ^13^C-NMR (CDCl_3_) δ 24.0 (d, *J* = 4.8, CH_3_), 24.1 (d, *J* = 4.3, CH_3_), 67.2 (d, *J* = 5.5, OCH_2_^a^), 71.5 (d, *J* = 6.0, OCH^a^), 128.0 (C_2_^b^), 128.7 (C_4_), 128.8 (C_3_^b^) 135.9 (d, *J* = 6.3, C_1_), ^a,b^may be reversed; ^1^H-NMR (CDCl_3_) δ 1.33 (m, 6H, OCH_3_), 4.65–4.82 (m, 1H, OCH), 5.10 (d, *J* = 9.3, 2H, OCH_2_), 6.89 (d, *J* = 697.1, 1H, PH), 7.31–7.43 (m, 5H, ArH); [M + H]^+^ = 215.0838, C_10_H_16_O_3_P requires 215.0837.

*Benzyl butyl-H-phosphonate* (**4d**). Yield: 0.27 g (40%); ^31^P-NMR (CDCl_3_) δ 7.9; ^13^C-NMR (CDCl_3_) δ 13.6 (CH_3_), 18.8 (CH_3_C*H*_2_), 32.4 (d, *J* = 6.2, OCH_2_C*H*_2_), 65.7 (d, *J* = 6.2, OCH_2_), 67.3 (d, *J* = 5.6, OCH_2_), 128.1 (C_2_*), 128.75 (C_4_), 128.80 (C_3_*), 135.8 (d, *J* = 6.0, C_1_), *may be reversed; ^1^H-NMR (CDCl_3_) δ 0.91 (t, *J* = 7.4, 3H, CH_3_), 1.31–1.44 (m, 2H, CH_2_), 1.58-1.69 (m, 2H, CH_2_), 3.95–4.11 (m, 2H, OC*H*_2_CH_2_), 5.11 (d, *J* = 9.6, 2H, OC*H*_2_Ph), 6.87 (d, *J* = 699.4, 1H, PH), 7.32–7.43 (m, 5H, ArH); [M + H]^+^ = 229.0989, C_11_H_18_O_3_P requires 229.0994.

NMR spectra of products **4a**–**d** are shown in the Supplementary Materials.

The characterization data of the dialkyl phosphites **5** is summarized in Table 9.

## 4. Conclusions

The microwave-assisted direct esterification of phenyl-*H*-phosphinic acid, transesterification of the resulting alkyl phenyl-*H*-phosphinates, as well as that of dibenzyl phosphite was elaborated, and then, after optimization of the parameters (temperature and flow rate), these transformations were translated into continuous flow methods. While the direct esterifications were performed at 160–200 °C, the transesterifications of the ethyl phosphinate required somewhat higher temperatures up to 225 °C. Dibenzyl phosphite was more reactive, and already took part in alcoholyses at 80–145 °C. The esterifications and transesterifications proved to be more productive in the flow embodiment. Regarding the esterifications, while the flow preparation may give ca. 0.75 g ester/30 min, the batch method affords only ca. 0.10 g product after the same time. In the optimized cases, esters **2** were obtained in yields above 75%. The two-step alcoholysis of dibenzyl phosphite involves an intermediate **4** with two different alkyl groups prepared in moderate yields that, among other uses, may be important precursors for optically active reagents in the Hirao reaction or the Kabachnik–Fields condensation, which will be further studied by us in future work.

## Figures and Tables

**Table 1:**
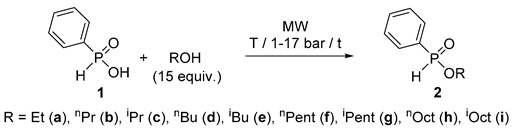
Direct esterification of phenyl-*H*-phosphinic acid (**1**) in a batch MW reactor.

Entry	R	IL	T (°C)	t (min)	Conversion * (%)	Yield (%)	Product	Ref.
1	Et	–	160	60	100	80	**2a**	[20]
2	Et	10% [bmim][PF_6_]	140	30	100	94	**2a**	[26]
3	^n^Pr	–	160	60	100	73	**2b**	[20]
4	^n^Pr	10% [bmim][PF_6_]	160	30	100	84	**2b**	
5	^i^Pr	–	180	120	58	48	**2c**	[20]
6	^i^Pr	10% [bmim][PF_6_]	180	120	96	80	**2c**	
7	^n^Bu	–	160	60	100	85	**2d**	[20]
8	^n^Bu	–	180	30	100	90	**2d**	
9	^n^Bu	–	200	10	100	89	**2d**	
10	^n^Bu	10% [bmim][PF_6_]	140	30	100	94	**2d**	[26]
11	^i^Bu	–	160	60	100	75	**2e**	[20]
12	^i^Bu	10% [bmim][PF_6_]	140	30	100	93	**2e**	[26]
13	^n^Pent	–	190	30	100	89	**2f**	[26]
14	^n^Pent	10% [bmim][PF_6_]	140	30	100	92	**2f**	[26]
15	^i^Pent	–	190	30	100	87	**2g**	
16	^i^Pent	10% [bmim][PF_6_]	150	30	100	94	**2g**	
17	^n^Oct	–	180	30	100	84	**2h**	[26]
18	^n^Oct	10% [bmim][PF_6_]	140	30	100	88	**2h**	[26]
19	^i^Oct	–	180	30	100	75	**2i**	
20	^i^Oct	10% [bmim][PF_6_]	150	30	100	82	**2i**	

* On the basis of relative ^31^P-NMR integrals.

**Table 2:**
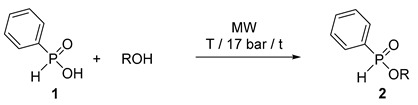
Direct esterification of phenyl-*H*-phosphinic acid (**1**) with different alcohols in a flow MW reactor in a concentration of 0.1 g/mL.

Entry	R	IL	T (°C)	V (mL/min)	Conversion ^a,b^ (%)	Yield ^c^ (%)	Product
1	^n^Bu	–	160	0.25	50	–	**2d**
2			160	0.15	54	–	**2d**
3			180	0.25	53	–	**2d**
4			180	0.15	64	–	**2d**
5			200	0.25	63	–	**2d**
6			200	0.15	72	–	**2d**
7		5% [bmim][PF_6_]	160	0.25	66	–	**2d**
8			160	0.15	72	–	**2d**
9			180	0.25	83	–	**2d**
10			180	0.15	95	–	**2d**
11			200	0.25	100	81	**2d**
12	Et	5% [bmim][PF_6_]	160 ^d^	0.15	65 ^e^	–	**2a**
13	^n^Pr	5% [bmim][PF_6_]	160	0.15	71	63	**2b**
14	^i^Pr	5% [bmim][PF_6_]	180	0.15	68	–	**2c**
15	^i^Bu	5% [bmim][PF_6_]	160	0.15	100	91	**2e**
16	^n^Pent	5% [bmim][PF_6_]	180	0.15	100	–	**2f**
17			190	0.25	100	85	**2f**
18	^i^Pent	5% [bmim][PF_6_]	180	0.15	97	–	**2g**
19			200	0.25	100	90	**2g**
20	^n^Oct	5% [bmim][PF_6_]	180	0.15	100	82	**2h**
21			190	0.25	100	84	**2h**
22	^i^Oct	5% [bmim][PF_6_]	180	0.15	100	86	**2i**
23			190	0.25	100	85	**2i**

^a^ On the basis of relative ^31^P-NMR integrals; ^b^ After reaching the steady state; ^c^ After an operation of 45 or 75 min belonging to 0.25 mL/min and 0.15 mL/min, respectively; ^d^ The comparative thermal experiment led to a conversion of 35%; ^e^ Recycling this mixture, and reacting under the same conditions, the final conversion was 100%. **2a** was isolated in a yield of 79%.

**Table 3:**
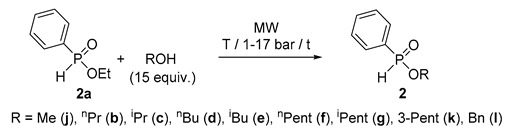
Transesterification of ethyl-phenyl-*H*-phosphinate (**2a**) in a batch MW reactor.

Entry	R	T (°C)	t (min)	Conversion * (%)	Yield (%)	Product
1	Me	120	180	93	79	**2j**
2	Me	140	120	89	74	**2j**
3	^n^Pr	180	60	97	83	**2b**
4	^i^Pr	180	120	89	74	**2c**
5	^n^Bu	160	135	100	90	**2d**
6	^n^Bu	180	40	100	89	**2d**
7	^i^Bu	160	135	100	85	**2e**
8	^n^Pent	180	40	100	91	**2f**
9	^i^Pent	190	40	100	88	**2g**
10	3-Pent	190	45	95	80	**2k**
11	Bn	180	60	100	90	**2l**

* On the basis of relative ^31^P-NMR integrals.

**Table 4:**
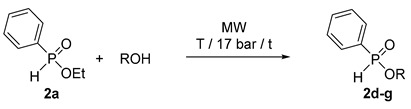
Transesterification of **2a** with n-butanol in a flow MW reactor in a concentration of 0.1 g/mL.

Entry	R	T (°C)	V (mL/min)	Conversion ^a,b^ (%)
1	^n^Bu (**d**)	180	0.25	53
2	^n^Bu (**d**)	180	0.15	62
3	^n^Bu (**d**)	200	0.25	71
4	^n^Bu (**d**)	200	0.15	84
5	^n^Bu (**d**)	220	0.25	81
6	^n^Bu (**d**)	220	0.15	94
7	^n^Bu (**d**)	225	0.15	100 ^c^
8	^i^Bu (**e**)	225	0.15	100 ^d^
9	^n^Pent (**f**)	220	0.15	100 ^e^
10	^i^Pent (**g**)	220	0.15	100 ^f^

^a^ On the basis of relative ^31^P-NMR integrals; ^b^ After reaching the steady state; ^c^ Yield of **2d**: 85%; ^d^ Yield of **2e**: 84%; ^e^ Yield of **2f**: 82%; ^f^ Yield of **2g**: 89%; ^c–f^ After an operation of 1 h.

**Table 5:**
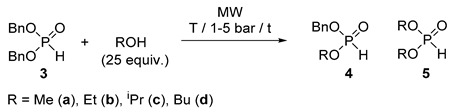
Alcoholysis of dibenzyl phosphite (**3**) in a batch MW reactor.

Entry	R	T (°C)	t (h)	Composition ^a^ (%)			Yield (%)	Product
				3	4	5		
**1**	Me	80	3 ^b^	26	57	17	49	**4a**
**2**		100	2	6	38	56	–	
**3**		120	0.5	26	54	20	47	**4a**
**4**		120	1.5	3	27	70	–	
**5**		120	2.5 ^b^	0	13	87	72	
**6**	Et	80	3 ^b^	49	50	1	–	
**7**		100	0.5	65	33	2	–	
**8**		100	0.5 ^c^	6	36	58	–	
**9**		100	2	11	64	25	59	**4b**
**10**		120	0.5	23	68	9	58	**4b**
**11**		120	1	9	51	40	–	
**12**		120	4 ^b^	0	11	89	75	
**13**	^i^Pr	100	2	35	60	5	51	**4c**
**14**		120	3 ^b^	4	33	63	–	
**15**		130	2.5	2	46	52	–	
**16**		130	5	0	5	95	80	
**17**	Bu	100 ^d^	2	8	61	31	52	**4d**
**18**		120	0.5	22	54	24	–	
**19**		120	1.5	0	29	71	–	
**20**		120	4	0	6	94	82	

^a^ On the basis of relative ^31^P-NMR integrals. DMSO-*d_6_* was used to ensure better separation of the signals; ^b^ No change of further irradiation; ^c^ In the presence of 20% [bmim]PF_6_; ^d^ The comparative thermal experiment led to a composition of 27% (**3**), 59% (**4d**), 14% (**5d**); the shaded percentage values refer to the maximum ratios.

**Table 6:**
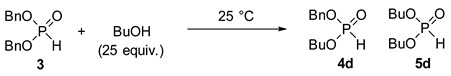
Alcoholysis of dibenzyl phosphite (**3**) at room temperature.

Entry	t (days)	Composition * (%)		
		3	4d	5d
**1**	1	98	2	0
**2**	3	89	11	0
**3**	5	84	16	0
**4**	7	82	20	2
**5**	10	71	28	1
**6**	14	64	35	1
**7**	18	54	44	2
**8**	24	36	57	7
**9**	31	25	62	13
**10**	38	16	67	17

* On the basis of relative ^31^P NMR integrals. DMSO-*d_6_* was used to ensure better separation of the signals.

**Table 7:**
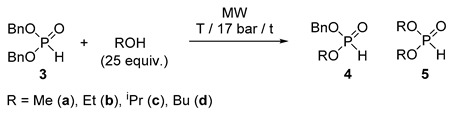
Continuous flow MW-assisted alcoholysis of dibenzyl phosphite.

Entry	R	V (mL/min)	T (°C)	Composition ^a,b^ (%)			Yield ^c^ (%)	Product
				3	4	5		
**1**	Me	0.25	110	24	52	24	44	**4a**
**2**		0.25	120	17	44	39	–	
**3**		0.15	135	5	23	72	–	
**4**	Et	0.25	120	28	48	24	41	**4b**
**5**		0.15	120	20	40	40	–	
**6**		0.25	135	17	36	47	–	
**7**		0.15	135	7	27	66	–	
**8**	^i^Pr	0.25	120	49	48	3	39	**4c**
**9**		0.15	145	18	36	46	–	
**10**	Bu	0.25	120 ^d^	30	47	23	40	**4d**
**11**		0.15	135	8	34	58	–	

^a^ On the basis of relative ^31^P-NMR integrals. DMSO-*d_6_* was used to ensure better separation of the signals; ^b^ After reaching the steady state; ^c^ After an operation of 30 or 45 min belonging to 0.25 mL/min and 0.15 mL/min, respectively; ^d^ Comparative thermal experiment at 120 °C after a steady state operation led to a composition of 49% (**3**), 47% (**4d**), 4% (**5d**).

**Table 8:** ^31^P-NMR and HRMS data for phosphinates **2**.

Product	R	δ_P_ (CDCl_3_)	δ_P_[lit]	[M + H]^+^_found_	[M + H]^+^_requires_	Formula
**2a**	Et	24.7	25.7 [30]	171.0569	171.0575	C_8_H_11_O_2_P
**2b**	^n^Pr	24.8	24.9 [20]	185.0725	185.0731	C_9_H_13_O_2_P
**2c**	^i^Pr	22.5	22.3 [20]	185.0726	185.0731	C_9_H_13_O_2_P
**2d**	^n^Bu	24.9	25.3 [30]	199.0881	199.0888	C_10_H_15_O_2_P
**2e**	^i^Bu	24.9	25.0 [20]	199.0881	199.0888	C_10_H_15_O_2_P
**2f**	^n^Pent	25.6	25.7 [23]	213.1037	213.1044	C_11_H_18_O_2_P
**2g**	^i^Pent	27.7	25.7 [23]	213.1042	213.1044	C_11_H_18_O_2_P
**2h**	^n^Oct	25.1	25.0 [23]	255.1517	255.1514	C_14_H_24_O_2_P
**2i**	^i^Oct	25.1	25.2 [23]	255.1516	255.1514	C_14_H_24_O_2_P

**Table 9:** ^31^P NMR and HRMS data for the known compounds **5a**–**d** prepared.

Compound	δ_P_ (CDCl_3_)	δ_P_[lit]	[M + H]^+^_found_	[M + H]^+^_requires_	Formula
**5a**	8.6	8.5 [32]	111.0213	111.0211	C_2_H_8_O_3_P
**5b**	7.8	7.9 [33]	139.0527	139.0524	C_4_H_12_O_3_P
**5c**	1.8	1.9 [32]	167.0837	167.0837	C_6_H_16_O_3_P
**5d**	8.6	8.4 [33]	195.1149	195.1150	C_8_H_20_O_3_P

## Supplementary Materials

Representative examples for the calculation of the conversions in the esterifications and transesterifications, as well as ^31^P-, ^13^C- and ^1^H-NMR spectra for the new alkyl, alkyl’ phosphites.

## References

[1] Gutmann B., Cantillo D., Kappe C.O. (2015). Continuous-flow technology—A tool for the safe manufacturing of active pharmaceutical ingredients. Angew. Chem. Int. Ed..

[2] Barham J.P., Koyama E., Norikane Y., Ohneda N., Yoshimura T. (2019). Microwave flow: A perspective on reactor and microwave configurations and the emergence of tunable single--mode heating toward large—Scale applications. Chem. Rec..

[3] Darvas F., Hessel V., Dorman G. (2014). Flow Chemistry.

[4] Dąbrowska S., Chudoba D., Wojnarowicz J., Łojkowski D. (2018). Current trends in the development of microwave reactors for the synthesis of nanomaterials in laboratories and industries: A Review. Crystals.

[5] Horikoshi S., Schiffmann R.F., Fukushima J., Serpone N. (2018). Microwave Chemical and Materials Processing.

[6] Cravotto G., Carnaroglio D. (2017). Microwave Chemistry.

[7] Keglevich G. (2016). Milestones in Microwave Chemistry—SpringerBriefs in Molecular Science.

[8] Keglevich G., Mucsi Z., Cravotto G., Carnaroglio D. (2017). Interpretation of the rate enhancing effect of microwaves. Microwave Chemistry.

[9] Tajti Á., Tóth N., Bálint E., Keglevich G. (2018). Esterification of benzoic acid in a continuous flow microwave reactor. J. Flow Chem..

[10] Bálint E., Tajti Á., Tóth N., Keglevich G. (2018). Continuous flow alcoholysis of dialkyl H-phosphonates with aliphatic alcohols. Molecules.

[11] Bálint E., Tajti Á., Keglevich G. (2019). Application of the microwave technique in continuous flow processing of organophosphorus chemical reactions. Materials.

[12] Keaveney S.T., Haines R.S., Harper J.B., Wang Z. (2017). Reaction in ionic liquids. Encyclopedia of Physical Organic Chemistry.

[13] Vekariya R.L. (2017). A review of ionic liquids: Applications towards catalytic organic transformations. J. Mol. Liq..

[14] Rádai Z., Kiss N.Z., Keglevich G. (2018). An overview of the applications of ionic liquids as catalysts and additives in organic chemical reactions. Curr. Org. Chem..

[15] Quin L.D. (2000). A guide to Organophosphorus Chemistry.

[16] Kiss N.Z., Keglevich G. (2014). An overview of the synthesis of phosphinates and phosphinic amides. Curr. Org. Chem..

[17] Keglevich G., Allen D.W., Loakes D., Tebby J.C. (2019). Phosphine chalcogenides. Specialist Periodical Reports on Organophosphorus Chemistry.

[18] Henyecz R., Keglevich G. (2019). New developments on the Hirao reactions, especially from “green” point of view. Curr. Org. Synth..

[19] Keglevich G., Bálint E. (2012). The Kabachnik–Fields reaction: Mechanism and synthetic use. Molecules.

[20] Kiss N.Z., Ludányi K., Drahos L., Keglevich G. (2009). Novel synthesis of phosphinates by the microwave-assisted esterification of phosphinic acids. Synth. Commun..

[21] Keglevich G., Kiss N.Z., Mucsi Z., Körtvélyesi T. (2012). Insights into a surprising reaction: The microwave-assisted direct esterification of phosphinic acids. Org. Biomol. Chem..

[22] Kiss N.Z., Böttger É., Drahos L., Keglevich G. (2013). Microwave-assisted direct esterification of cyclic phosphinic acids. Heteroatom Chem..

[23] Kiss N.Z., Mucsi Z., Böttger É., Drahos L., Keglevich G. (2014). A three-step conversion of phenyl-1H-phosphinic acid to dialkyl phenylphosphonates including two microwave-assisted direct esterification steps. Curr. Org. Synth..

[24] Kiss N.Z., Keglevich G. (2016). Microwave-assisted direct esterification of cyclic phosphinic acids in the presence of ionic liquids. Tetrahedron Lett..

[25] Kiss N.Z., Keglevich G. (2019). Direct esterification of phosphinic and phosphonic acids enhanced by ionic liquid additives. Pure Appl. Chem..

[26] Henyecz R., Kiss A., Mórocz V., Kiss N.Z., Keglevich G. (2019). Synthesis of phosphonates from phenylphosphonic acid and its monoesters. Synth. Commun..

[27] Bálint E., Tajti Á., Drahos L., Ilia G., Keglevich G. (2013). Alcoholysis of dialkyl phosphites under microwave conditions. Curr. Org. Chem..

[28] Tajti Á., Bálint E., Keglevich G. (2016). Synthesis of ethyl octyl α-aminophosphonate derivatives. Curr. Org. Synth..

[29] Lewkowski J., Moya M.R. (2017). The formation of dimethyl amino(pyrene-1-yl)methylphosphonates in the Kabachnik-Fields reaction with dibenzyl phosphite, pyrene-1-carboxaldehyde and a non-aromatic amine in methanol. Phosphorus Sulfur Silicon.

[30] Hewitt D.G. (1979). Organophosphorus compounds. II. Ethyl phenylphosphinate. Aust. J. Chem..

[31] Froneman M., Modro T.A. (1991). New synthesis of phosphorus and phosphoric acid esters. Synthesis.

[32] Ma L., Li G., Li L., Liu P. (2010). Synthesis and characterization of diethoxy phosphoryl chitosan. Int. J. Biol. Macromol..

[33] Li C., Wang Q., Zhang J.-Q., Ye J., Xie J., Xu Q., Han L.-B. (2019). Water determines the products: An unexpected Brønsted acid-catalyzed PO–R cleavage of P(III) esters selectively producing P(O)–H and P(O)–R compounds. Green Chem..

