# Supplementary material for: Continuous Flow Esterification of a H-Phosphinic Acid, and Transesterification of H-Phosphinates and H-Phosphonates under Microwave Conditions
Source: Molecules. 2020 Feb 7;25(3):719. doi: 10.3390/molecules25030719 (PMC7037008; doi:10.3390/molecules25030719)
Supplement: Supplementary file 1 [file molecules-25-00719-s001.pdf]

Supplementary Information

Continuous flow esterification of a *H*-phosphinic acid, and transesterification of *H*-phosphinates and *H*-phosphonates under MW conditions

Nóra Zsuzsa Kiss, Réka Henyecz and György Keglevich*

Department of Organic Chemistry and Technology, Budapest University of Technology and Economics, 1521 Budapest, Hungary

Corresponding author: Dr. György Keglevich, e-mail: gkeglevich@mail.bme.hu

Table of contents:

1) Representative examples for the calculation of the conversions in the esterifications and transesterifications ....pg. S1–S3

2) ^{31}P, ^{13}C and ^{1}H NMR spectra of the new dialkyl phosphites with different alkoxy groups (**4a-d**) ....pg. S4–S9

1) Representative examples for the calculation of the conversions in the esterifications and transesterifications

Conversions were obtained based on relative ^{31}P NMR integrals. Typical examples are illustrated below. However, conversions and compositions shown in Tables 1-7 are the results of 3 parallel experiments with a pointing error of <5%. Peaks listed in Tables 8 and 9 show the shifts of the purified compounds.

Direct esterification of phenyl-*H*-phosphinic acid (1) with butanol

Conversion: 63% (Table 2/Entry 5)

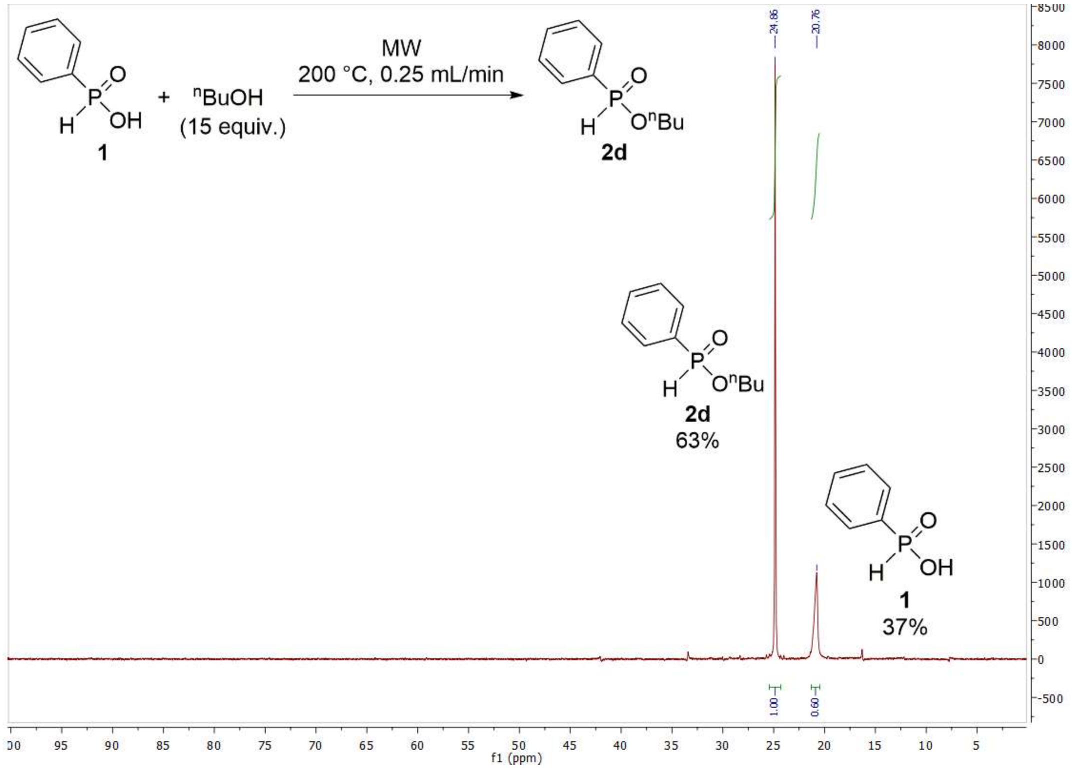

Conversion: 100% (Table 2/Entry 11)

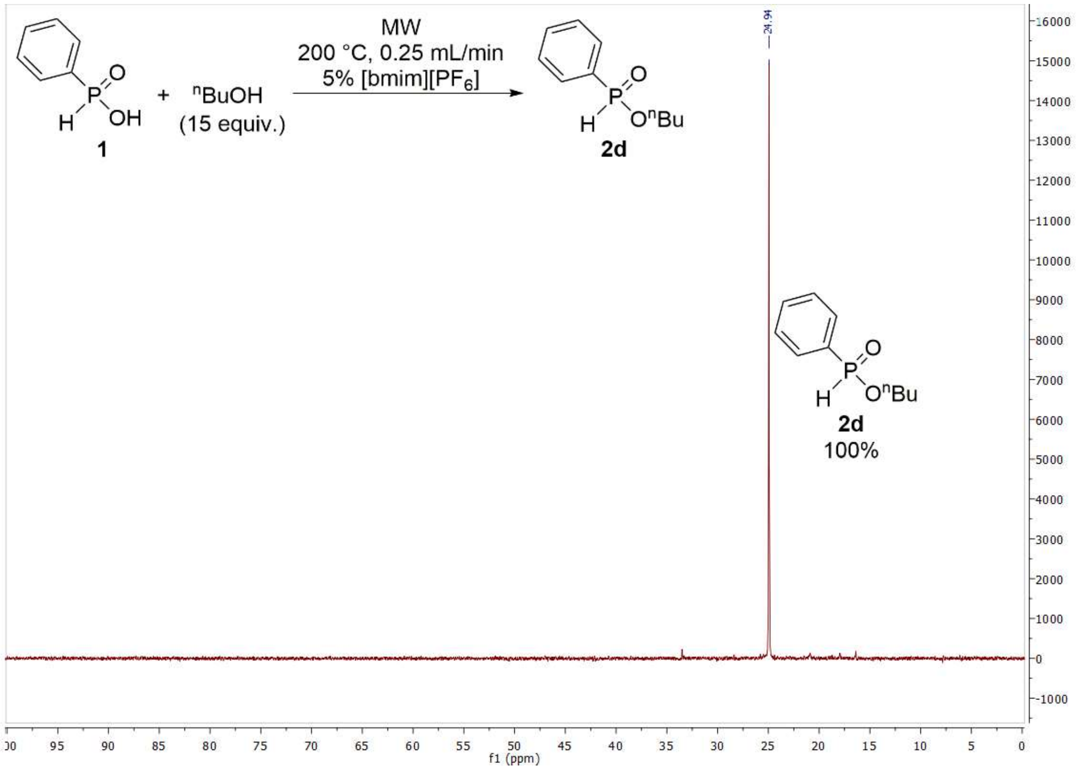

Transesterification of ethyl phenyl-*H*-phosphinate (2a)

Conversion: 89% (Table 3/Entry 7)

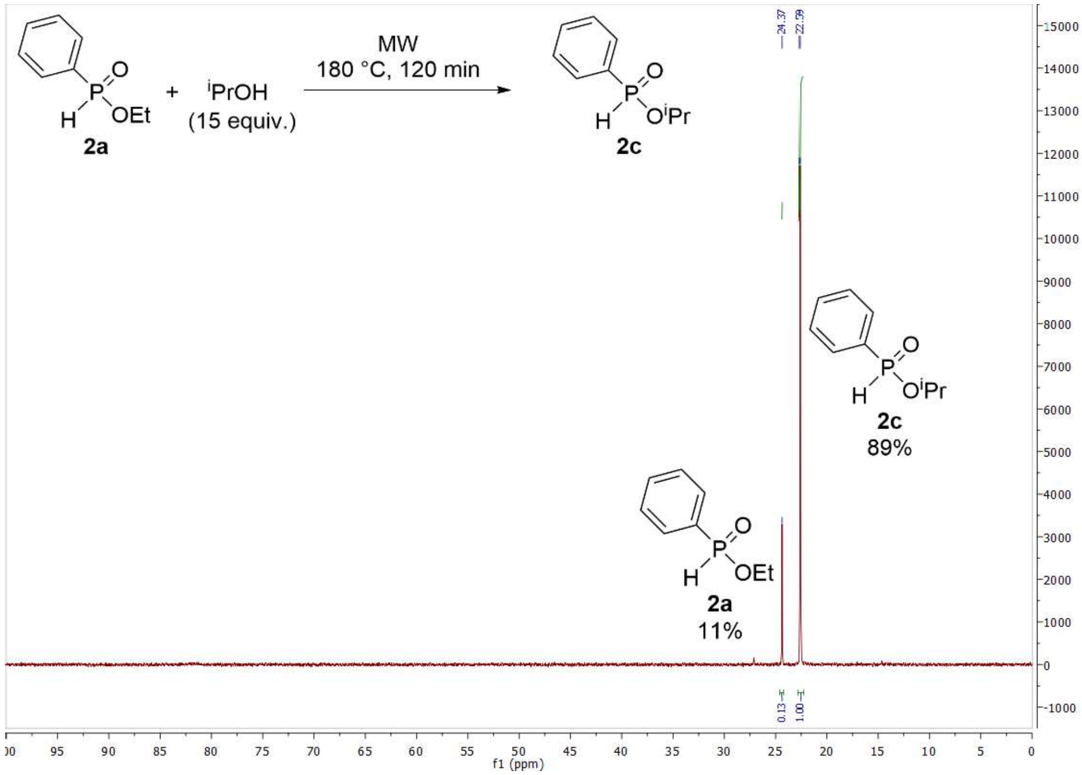

Transesterification of dibenzyl phosphite (3)

Composition: 35% (**3**), 60% (**4c**) and 5% (**5c**) (Table 5/Entry 13).

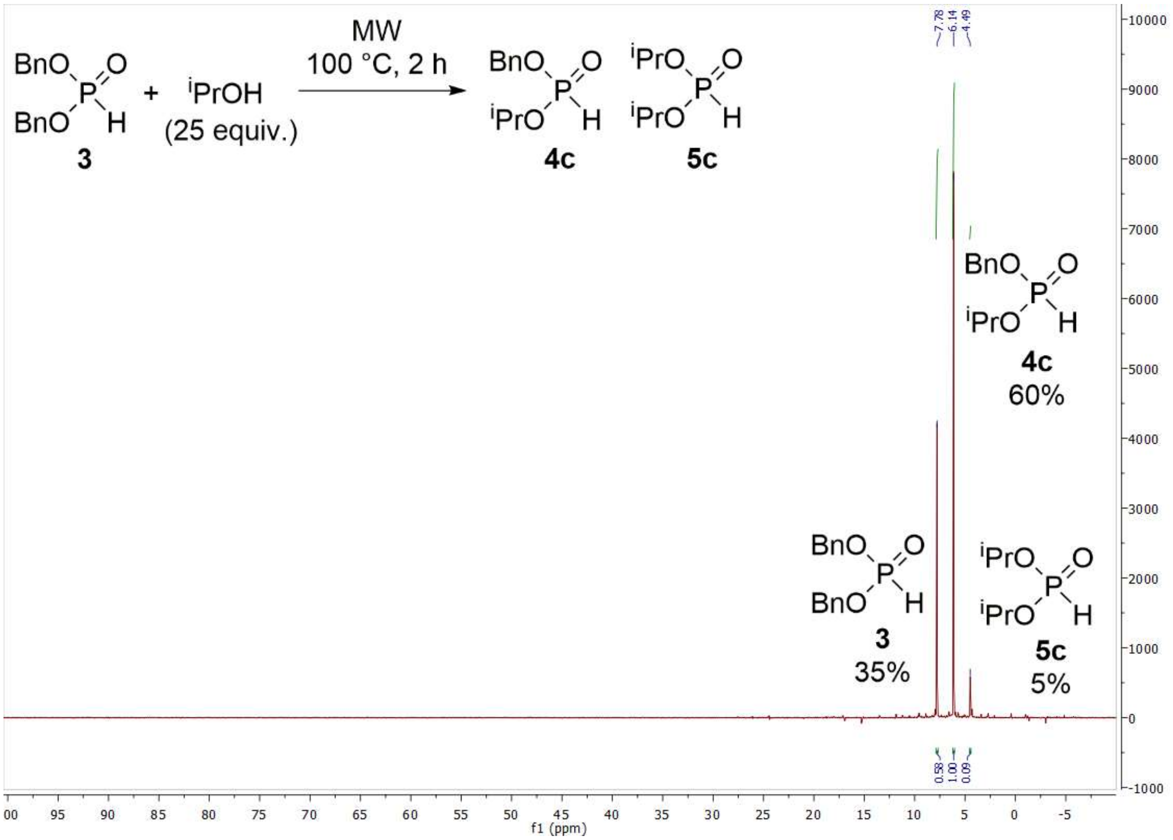

2) ^{31}P, ^{13}C and ^{1}H NMR spectra of the new dialkyl phosphites with different alkoxy groups (4a–d)

Benzyl methyl-*H*-phosphonate (**4a**)

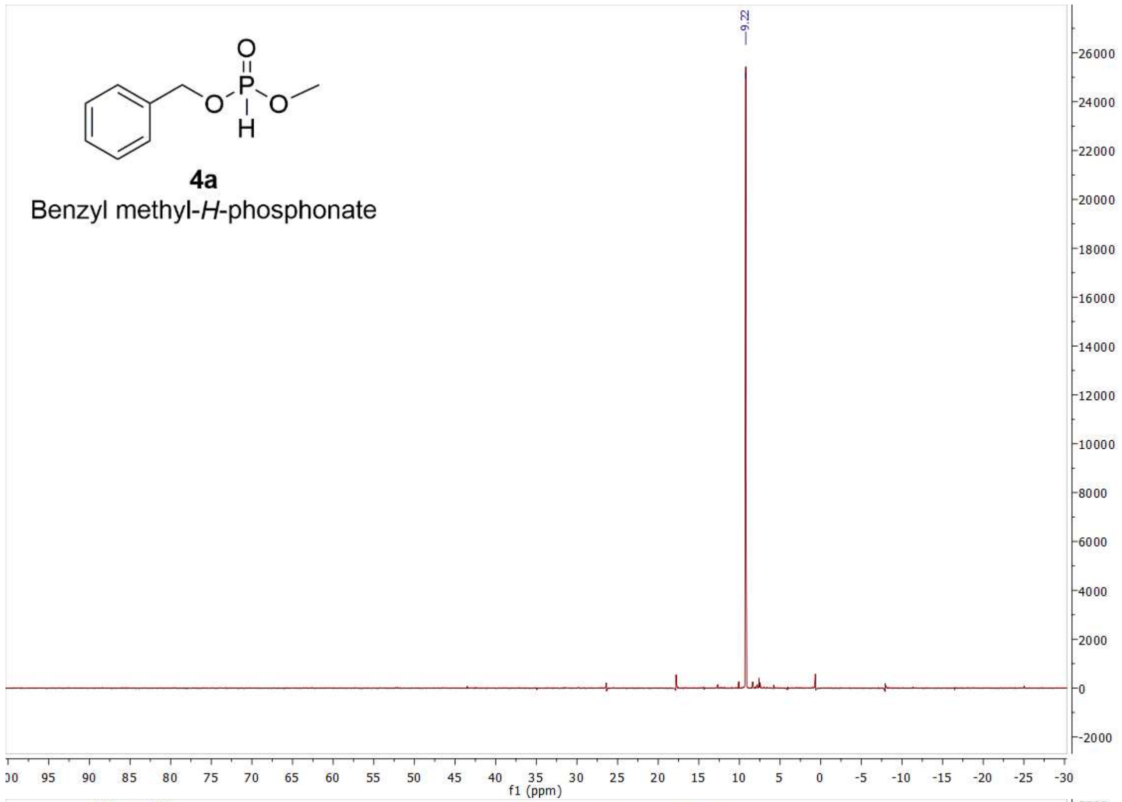

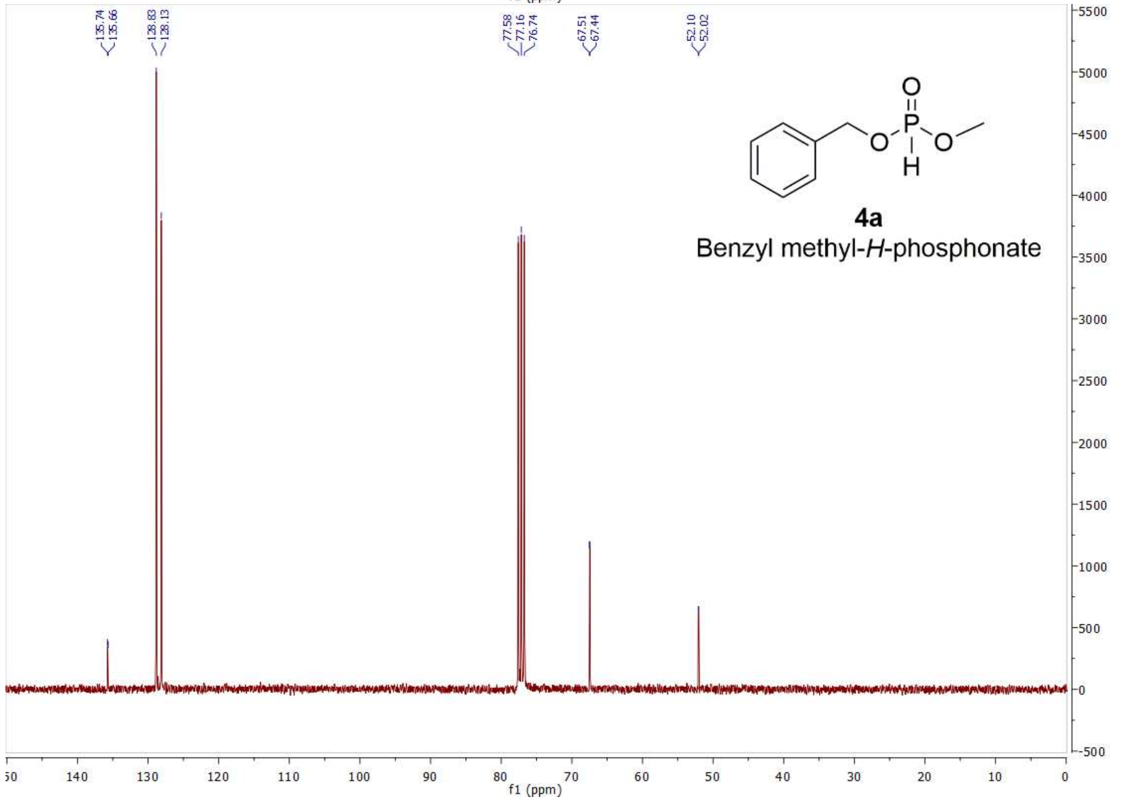

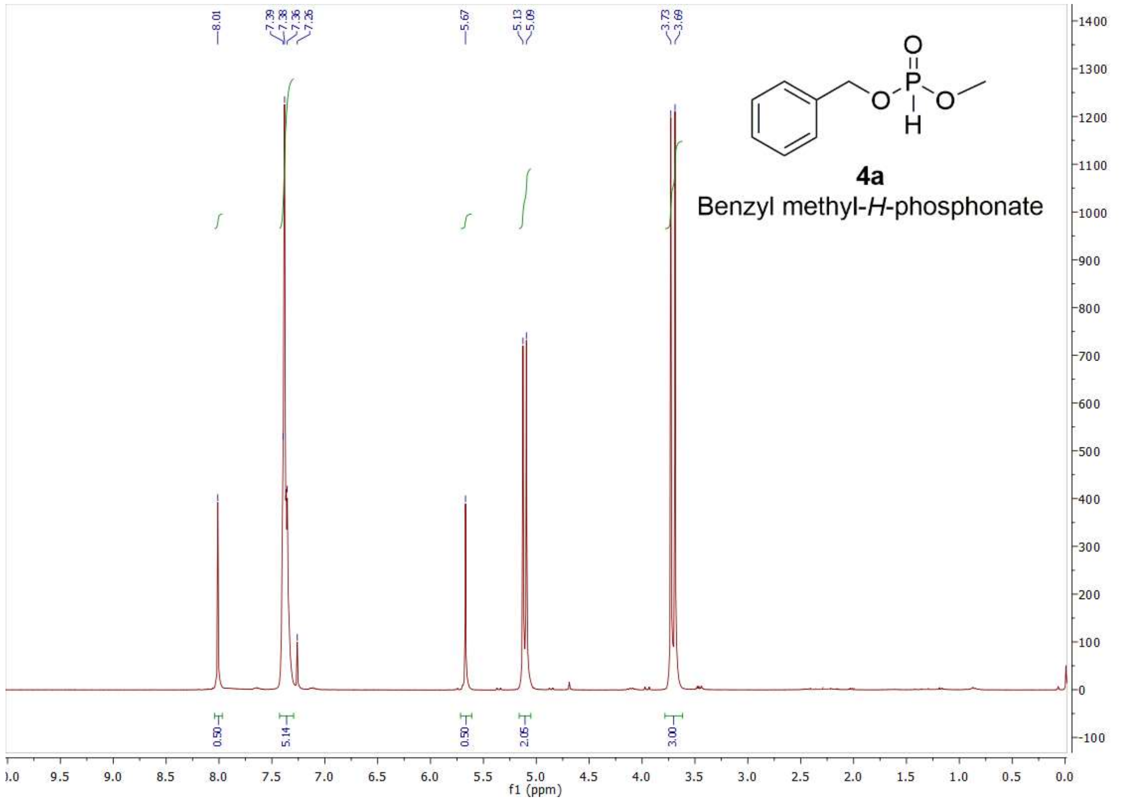

Benzyl ethyl-*H*-phosphonate (**4b**)

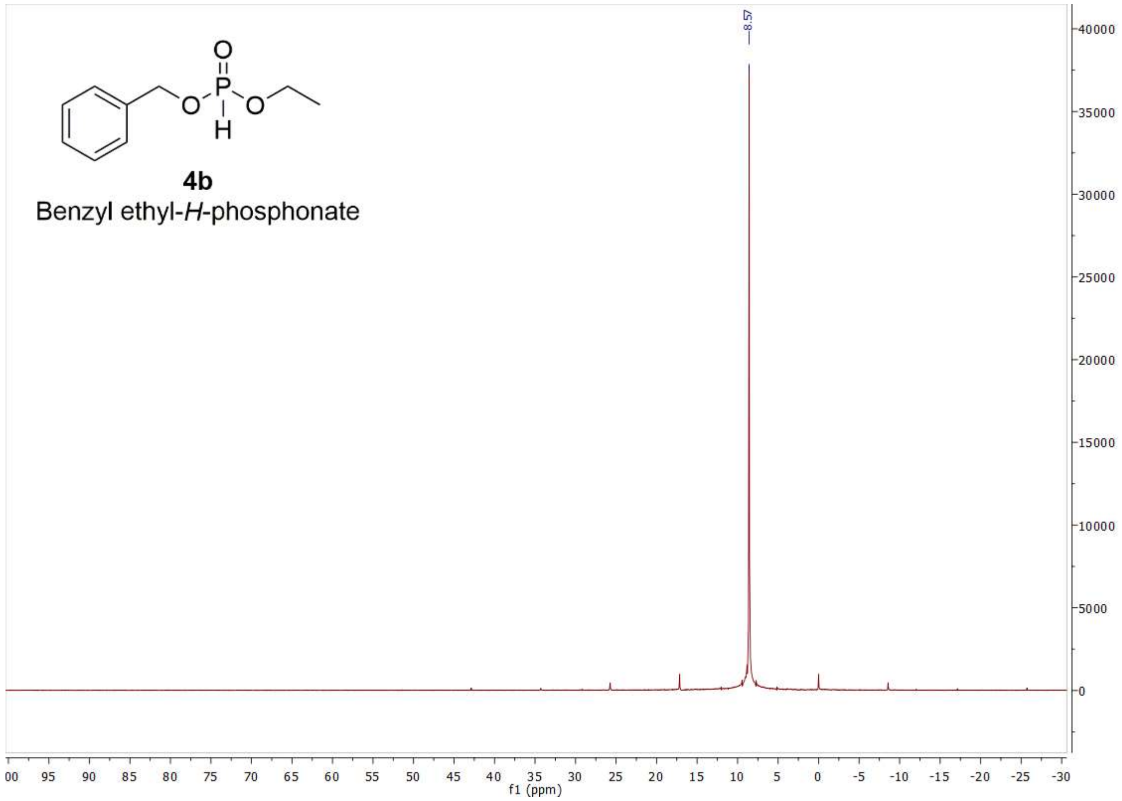

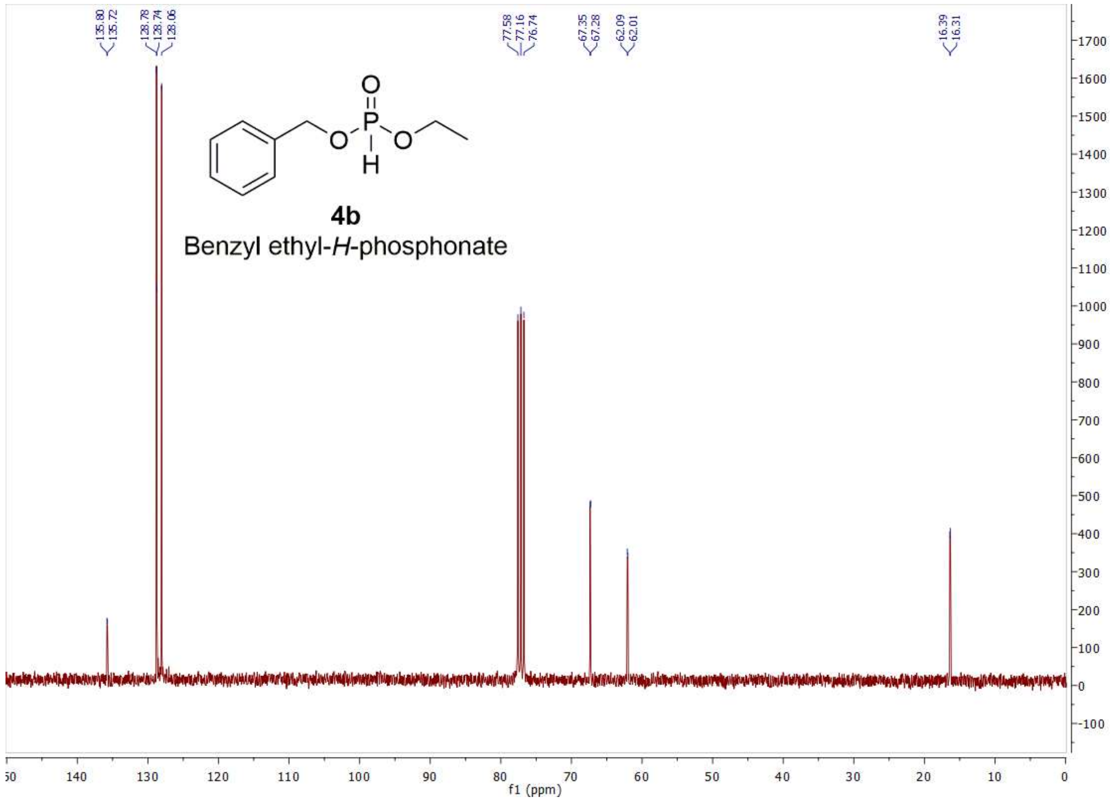
135.80
135.72
128.78
128.74
128.06
77.58
77.16
76.74
67.35
67.28
62.09
62.01
16.39
16.31
4b
Benzyl ethyl-*H*-phosphonate
f1 (ppm)

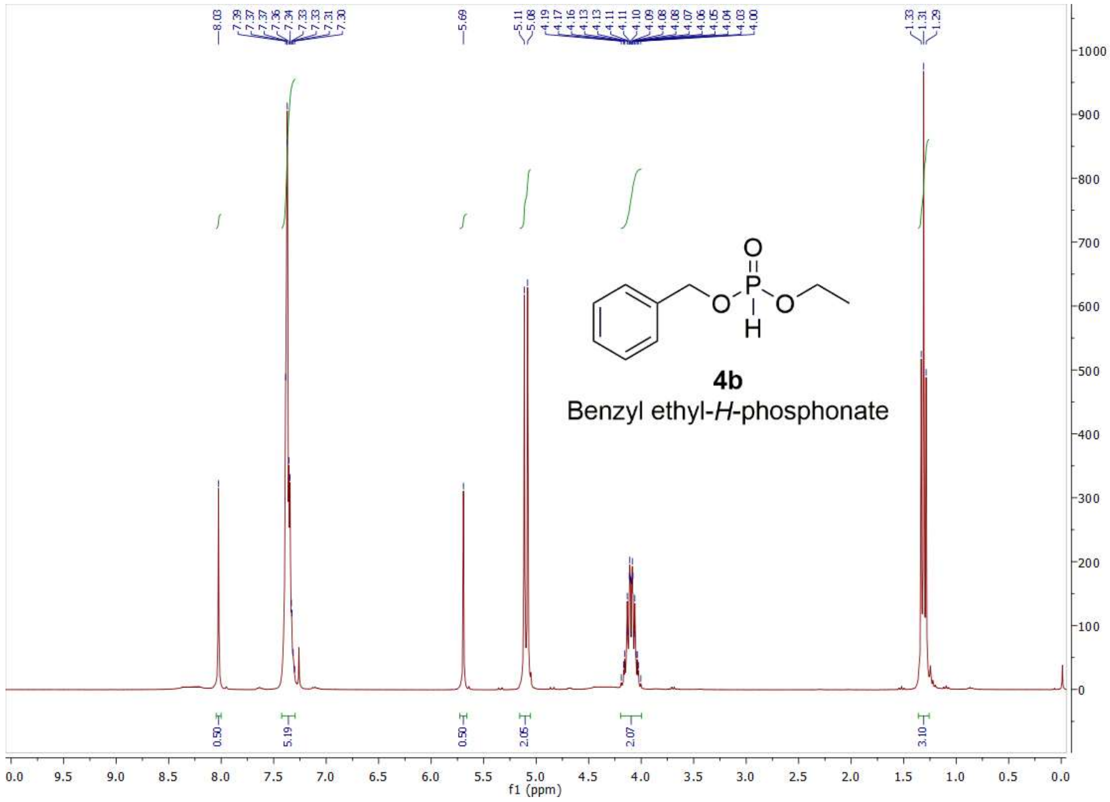
4b
Benzyl ethyl-*H*-phosphonate
f1 (ppm)

Benzyl isopropyl-*H*-phosphonate (**4c**)

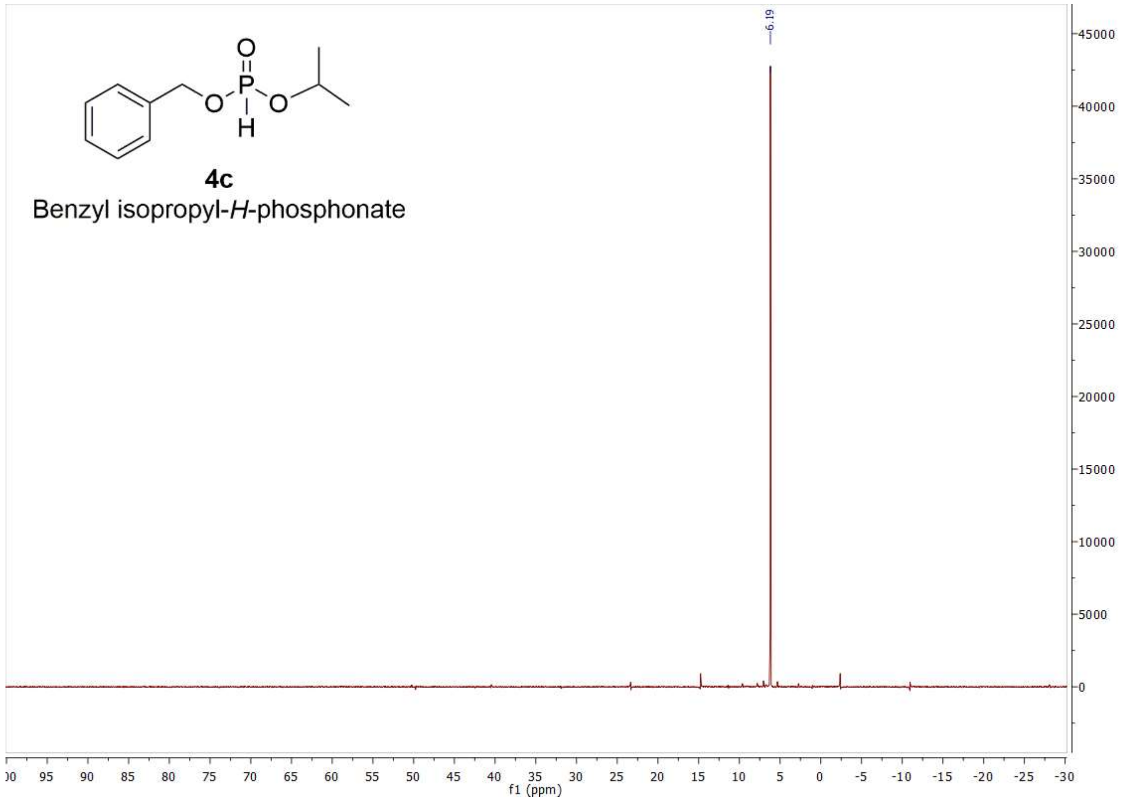

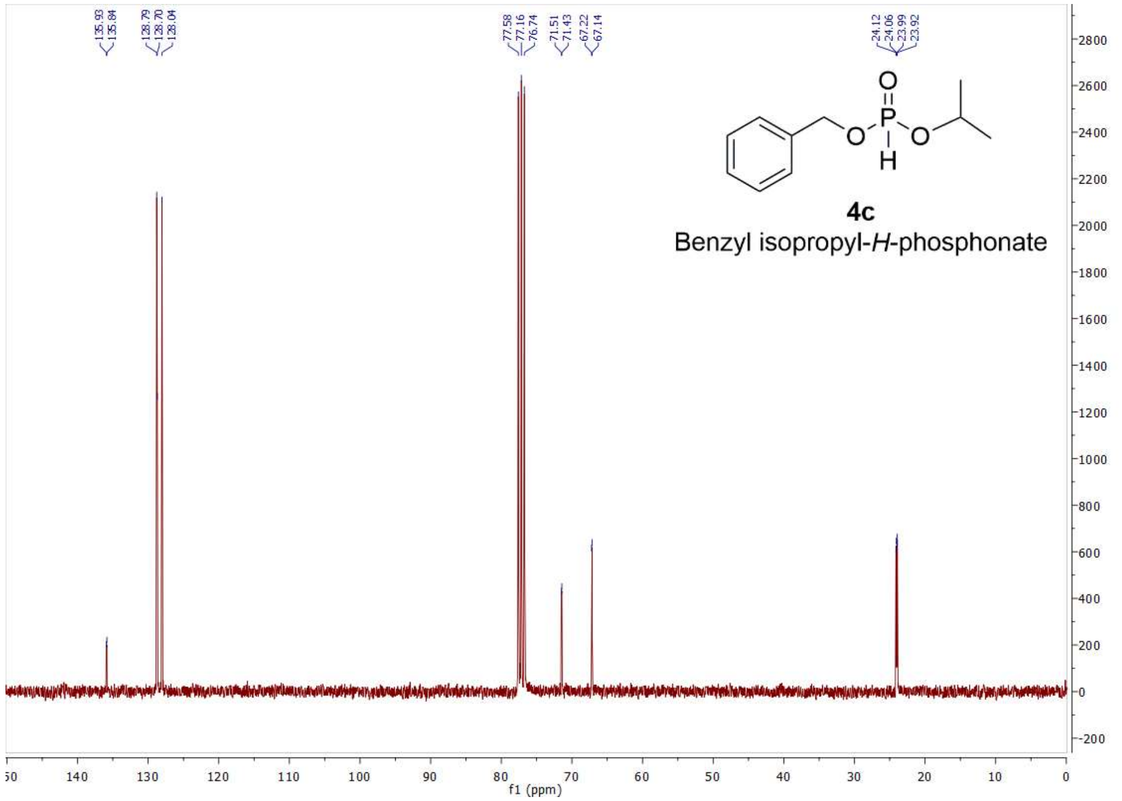

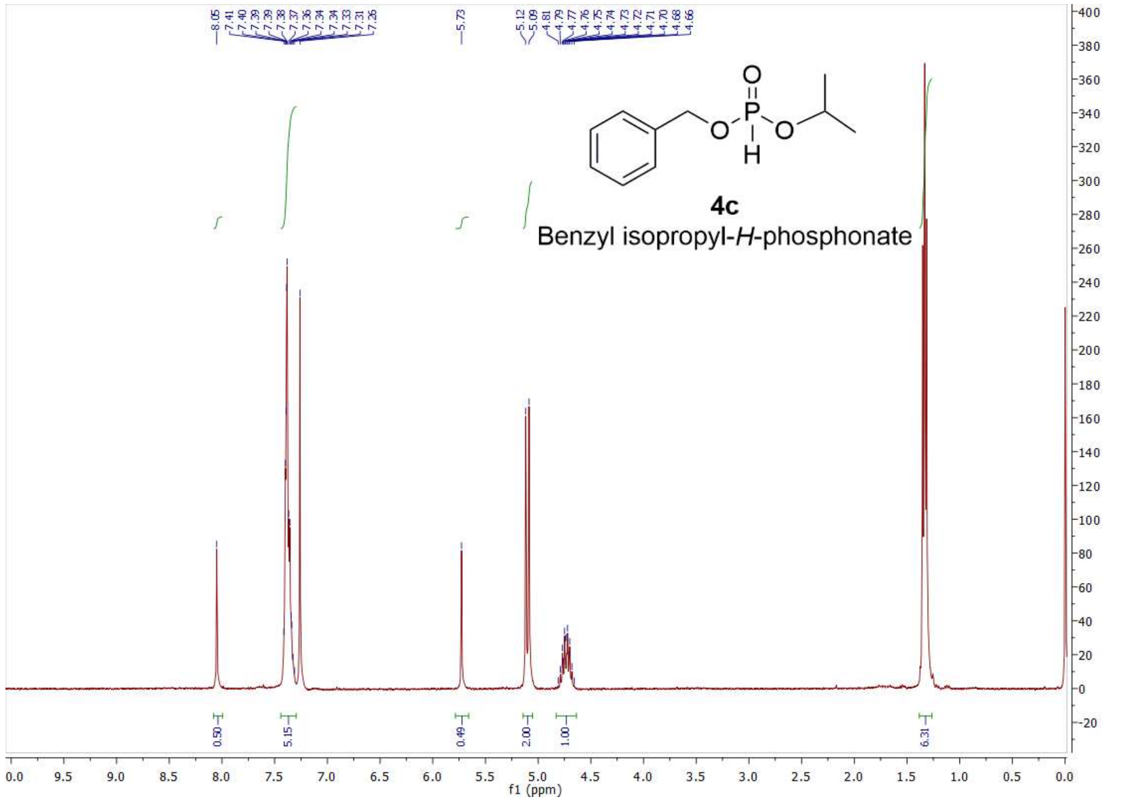

Benzyl butyl-*H*-phosphonate (**4d**)

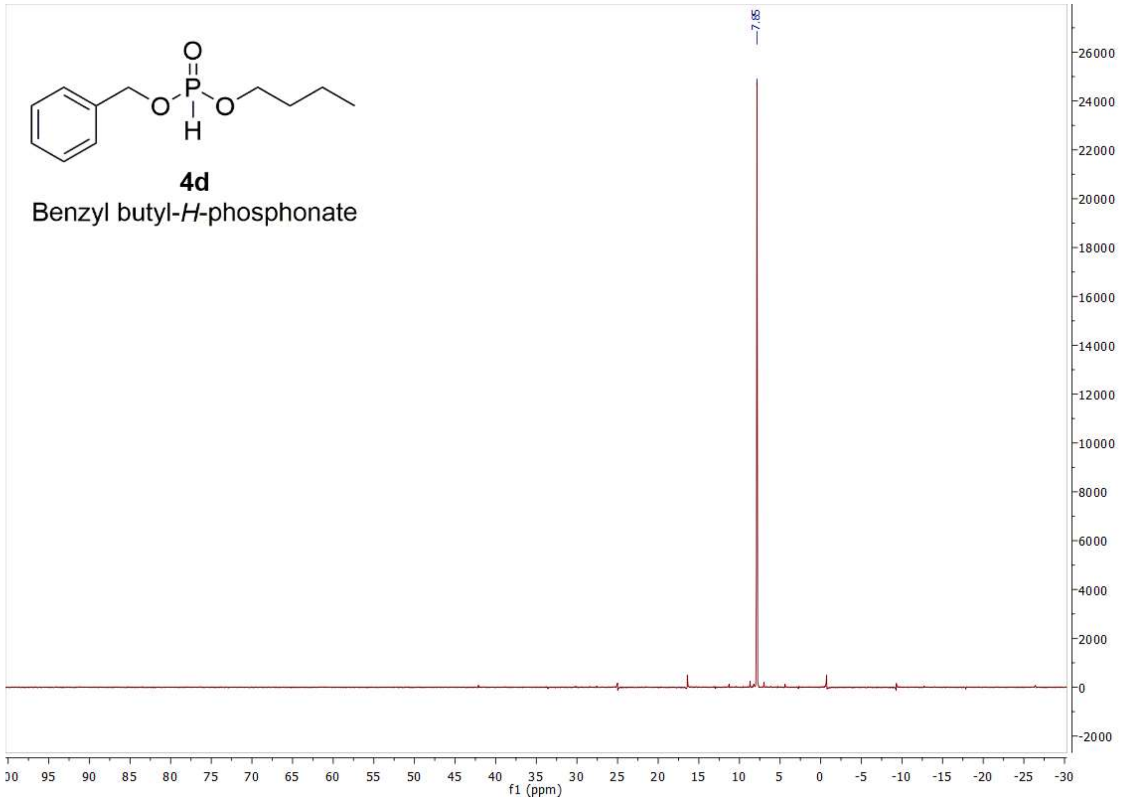

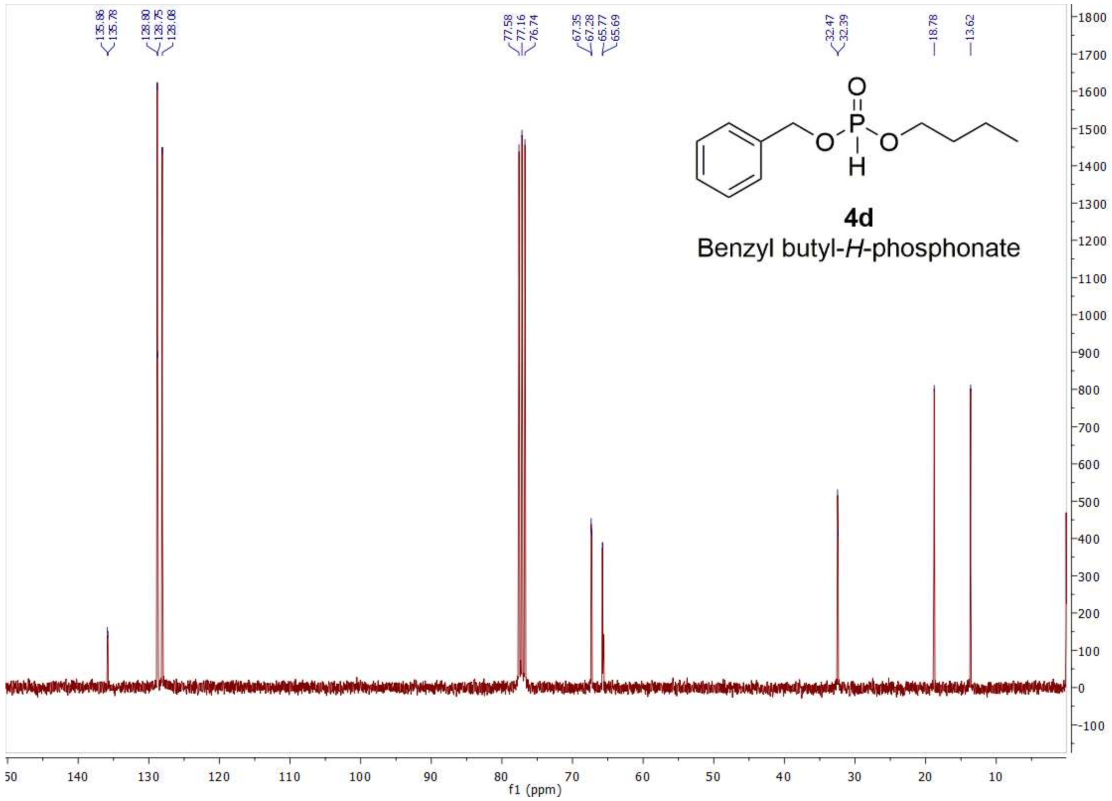
135.86
135.78
128.80
128.75
128.08
77.58
77.16
76.74
67.35
67.28
65.77
65.69
32.47
32.39
18.78
13.62
4d
Benzyl butyl-*H*-phosphonate
f1 (ppm)

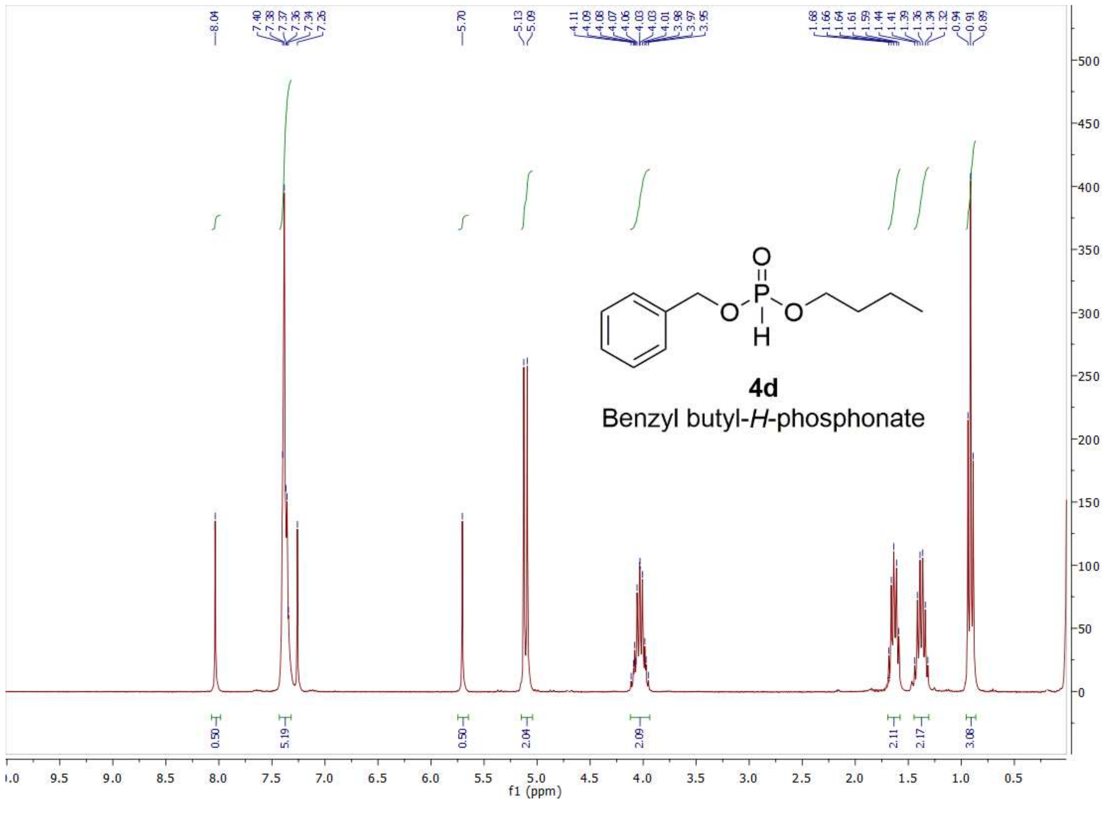
8.04
7.40
7.38
7.37
7.36
7.34
7.26
5.70
5.13
5.09
4.11
4.09
4.08
4.07
4.06
4.03
4.01
3.98
3.97
3.95
1.68
1.66
1.64
1.61
1.59
1.44
1.41
1.39
1.36
1.34
1.32
0.94
0.91
0.89
4d
Benzyl butyl-*H*-phosphonate
0.50
5.19
0.50
2.04
2.09
2.11
2.17
3.08
f1 (ppm)
